# Sore Gums or Throat

**Published:** 1869-02

**Authors:** 


Sore Gums or Throat.—Refined saltpetre is very useful in the treatment of these affections. Take a bit as big as a pea and let it slowly dissolve in the mouth, and from time to time repeat this and great relief will be experienced.

